# Introduction to the special issue on status epilepticus: neuronal injury, plasticity, and therapies; Celebrating the legacy of Dr. Claude G. Wasterlain

**DOI:** 10.1002/epi4.12724

**Published:** 2023-04-20

**Authors:** Jerome Engel Jr, Solomon L. Moshé, Astrid Nehlig, Denson G. Fujikawa, Raman Sankar, David E. Naylor, Andrey M. Mazarati, Claude G. Wasterlain

**Affiliations:** ^1^ David Geffen School of Medicine University of California Los Angeles Los Angeles California USA; ^2^ Albert Einstein College of Medicine Bronx New York USA; ^3^ University of Strasbourg Strasbourg France; ^4^ Neurology Service Veterans Administration Greater Los Angeles Healthcare System Los Angeles California USA

**Keywords:** Claude Wasterlain, epilepsy, status epilepticus

## 1 FOREWORD

**FIGURE 1:**
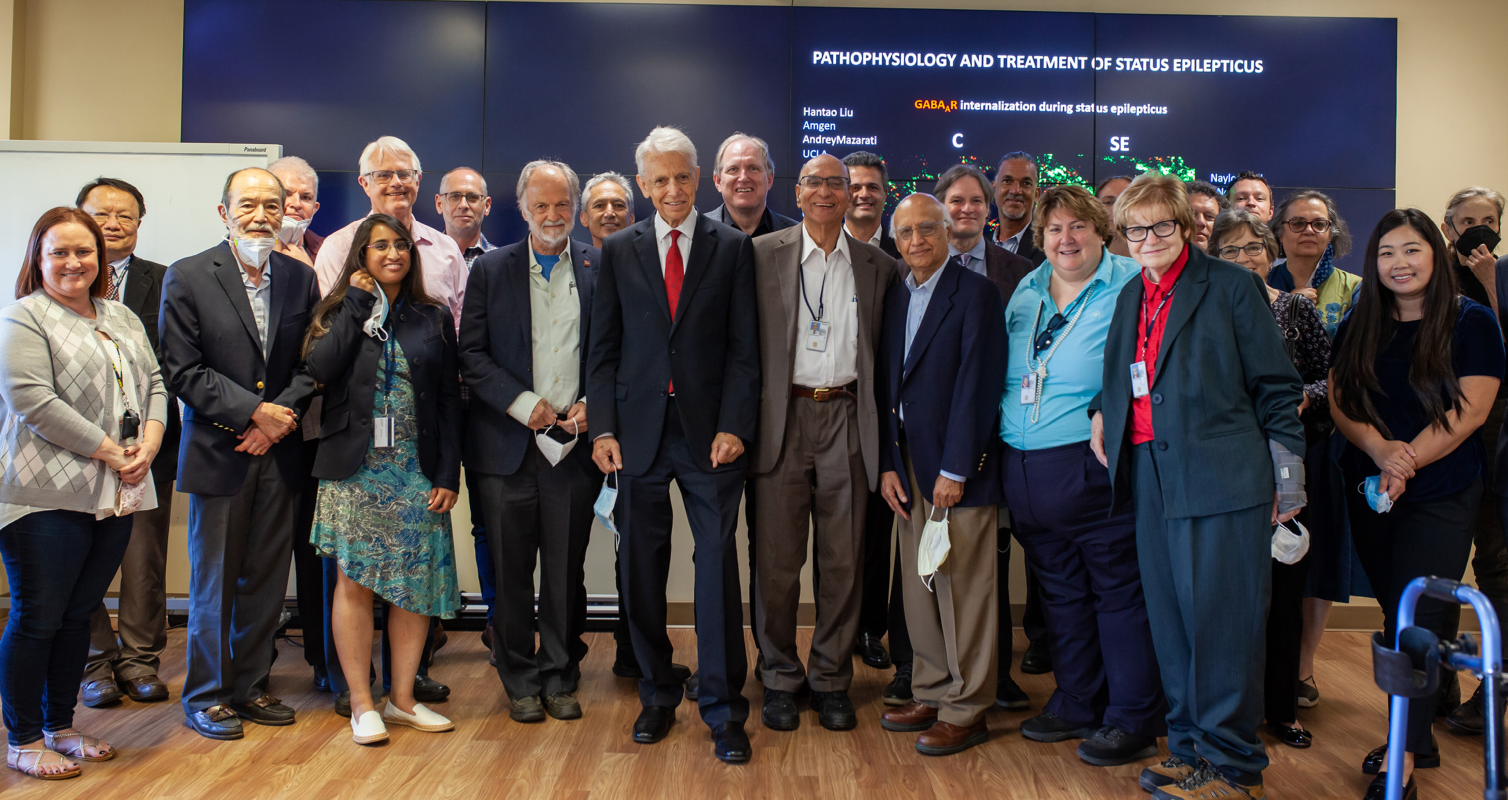
Dr. Claude G. Wasterlain (the tallest one, front and center) surrounded by friends and colleagues (*n* = 24; names available upon request). Symposium *Pathophysiology and treatment of status epilepticus*, West Los Angeles VA Medical Center, Los Angeles, California, July 31, 2021. *From the archive of David E. NaylorI*.

We are delighted to present this Special Issue of *Epilepsia Open* as a tribute to Dr. Claude G. Wasterlain, his many years of service and immense contributions to clinical and basic epileptology. We open this festschrift with the Introduction, where several of Claude's close friends and colleagues share their thoughts and memories. We also asked Claude to contemplate on his ways into, and life in Science. The main content is largely based on presentations given at the symposium *Pathophysiology and Treatment of Status Epilepticus*, held in honor of Claude at the West Los Angeles Veteran Administration Medical Center on July 31, 2021 (Figure 1). This collection of articles covers various topics pertinent to Status Epilepticus (which, along with Primitive Art and Impressionism is a subject particularly dear to Claude), including structural and functional perturbations, epileptogenesis, and treatment. We wish to thank the authors for taking time to contribute to this exciting project.

*Denson G. Fujikawa, MD, Andrey M. Mazarati, MD, PhD, David E. Naylor, MD, PhD, Raman Sankar, MD, PhD, Guest Editors*.

*P.S*. We would be remiss not to mention Belgium: the country that gave the world (in no particular order) beer, Victor Horta, fries, James Ensor, pralines, and Claude, forever deserves our utmost gratitude!

## 2 A WORD FROM JEROME ENGEL, JR., M.D.

### 2.1 Things you may not know about Claude Wasterlain

When I came to UCLA in November 1976, Claude was already here. As far as I was concerned, he had always been here, although he had only arrived 10 months earlier. To me, he was a constant that embodied UCLA Neurology and it is hard to imagine our department without him. Claude is my oldest friend in the University. Everyone knows about, and will discuss, Claude's clinical acumen, his administrative dexterity in maneuvering through the VA system, his seminal research contributions, especially in neonatal seizures and status epilepticus, earning him the Milken Award, the most prestigious award of the American Epilepsy Society, and the Gloor Award of the American Clinical Neurophysiology Society, his global involvement, for which he was named an Ambassador for Epilepsy by the International League against Epilepsy, and his consummate teaching prowess, with many former clinician–scientist trainees as his most important legacy. So, I would like to cover some facts most people may not know about Claude.

Claude comes from the coal country of southern Belgium, essentially the West Virginia of Europe. His people were all coal miners, but his grandfather was also a professional wrestler, perhaps explaining Claude's athletic skills on the tennis court and ski slopes. Claude's father shunned the mines and became an accountant. Given natural progression, there was never any doubt in his mother's mind that Claude would be the first in the family to attend University.

Claude was destined to end up in California because, as a child, he was infatuated with the American west, and cowboys. He had a cowboy hat his uncle gave him, actually a Canadian Mountie's hat that he put on for protection when he went outside to watch the bombing of a nearby railhead during World War II. He was also interested in poetry, won a poetry prize, and wanted to be a poet, but his mother convinced him to go to university to study pharmacy, a more stable occupation. After a year of pharmacy, spent mostly hanging out at pubs and playing foosball, he sought something more challenging and ended up in medicine. Claude credits his encounter with a patient with alexia without agraphia as committing him to a career in neurology.

Claude's subsequent success can largely be attributed to a secret super power. All super heroes have super powers, and Claude's is readily observable—we have all seen it, but few recognize it for what it is. I checked with Claude and he is OK with my revealing this information. At conferences large and small, Claude likes to sit in the front of the room. Eventually, he will fall asleep, then after several minutes, he will awaken and ask an incredibly perceptive question. It is clear that Claude can absorb information in his sleep. While most of us need to be awake to absorb information, Claude is actually better at absorbing and processing information in his sleep, explaining his encyclopedic knowledge.

Claude did his neurology residency at Cornell under Fred Plum, arguably the most respected, and certainly the most feared, American neurologist at the time. Claude tells of the time he fell asleep during morning rounds (obviously to better understand what was being demonstrated) and tumbled off his stool. Rather than being upset, Plum was pleased that a resident felt comfortable enough in his presence to fall asleep. The next morning, he offered Claude a faculty position.

Before beginning as faculty at Cornell, Claude spent 2 years back in Belgium where he obtained a MS in molecular biology. This was not merely a quest for more training, but necessary to satisfy Belgian draft requirements and avoid being sent to Vietnam; just as many of us discharged our military obligations during the Vietnam era as Research Associates at the National Institutes of Health for 2 years, while being commissioned in the Public Health Service. We were dubbed the yellow berets.

Claude returned to Cornell as a faculty member in 1969 when I was a neurology resident across town at Albert Einstein College of Medicine in the Bronx. I do not think we met at that time, but we all heard much about the young investigator at Cornell who was demonstrating the deleterious effects of neonatal seizures on rat brains at a time when these events in patients were considered benign and often not even treated. So, Claude was already famous when he went to UCLA, and I was very pleased to become his colleague. We have been friends ever since, and traveled the world together, with memorable side trips to places like Sri Lanka and Cappadocia between international meetings. My only question about the thousands of pleasant hours I have spent with Claude, academically and socially, concerns the fact that I cannot recall a single time that he fell asleep while I was talking. Does this mean that he never considered anything I ever said important enough to absorb and process it?

## 3 A WORD FROM SOLOMON L. MOSHÉ, M.D.

Back in 1977 when I started working on kindling in the developing brain, I did a literature search to see what kind of developmental studies had been done to study epileptogenesis and consequences of seizures. In contrast to the current interest in early‐life epilepsies, back then there were very few investigators interested in the topic: Caviness, Purpura, Vernadakis and Woodbury, Pavel Mares, and Claude Wasterlain.

Claude became my idol. He was the pioneer investigator studying the consequences of seizures in the developing brain of rats, rabbits, and monkeys. His studies showed, among other data, that 2 h of bicuculline or flurothyl seizures in 4‐day‐old rats, decreased the total forebrain content of DNA and protein 3 days after the seizures. Yet there was no evidence of necrosis in the hippocampus. Thirty days after the exposure to flurothyl, the long‐term effects of the 2 h episode of seizures on total forebrain content of DNA was dependent on the dose of flurothyl used to induced the status epilepticus: there was complete recovery with the low dose of flurothyl (and bicuculline) while there was partial recovery with a high dose of flurothyl. His studies seem to be misinterpreted a bit, as they were often quoted as evidence that the immature brain is prone to seizure‐induced injury. I chose a more optimistic view: the normal developing brain (at least in the rat) is more resistant to seizure‐induced injury and has a greater ability to recuperate. In the ensuing years, we had many opportunities to interact.

**FIGURE 2:**
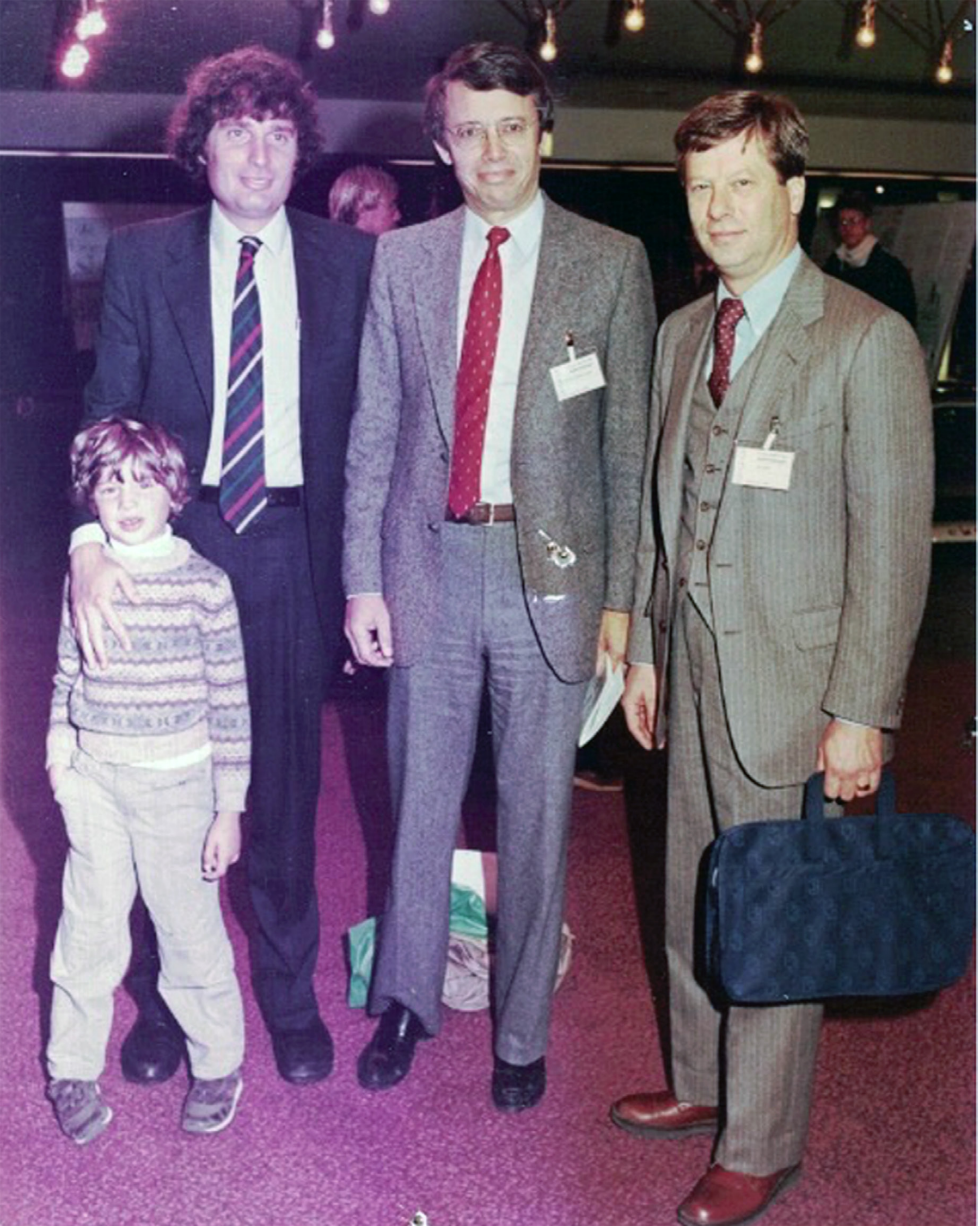
*Left to right*: Jared Moshé, Dr. Solomon L. Moshé, Dr. Claude G. Wasterlain and Dr. Alan Wyler. 15th International Epilepsy Congress, Hamburg, Germany, 1985. *From the archive of Solomon L. Moshé*.

But first, we had to meet. Our first encounter was in 1985. I went to the ILAE meeting in Hamburg as a co‐recipient of the Michael Prize award with Jeff Noebels. At that time, the registration fee was too high for my budget. As it was not covered by the Michael Prize committee, I did not register for the congress. However, again contrary to what is happening nowadays, anyone could go to the poster area. So I walked with my then 6‐year‐old son, Jared, looking at posters when suddenly I spotted Claude chatting with Alan Wyler. Here is my hero, in person. I walked over there, introduced my son and myself, and I asked to have my picture taken with him. Claude obliged (Figure 2). I felt that now I was a member of the club.

Subsequently, starting with the kindling symposia, I had the opportunity to interact with Claude on many occasions. His lectures were full of data although sometimes he was hard to follow as he tended to mumble, and his Belgium accent was interfering. I also noticed (and not for the last time) that during most of the conferences, Claude would appear to be asleep (he probably was), but when the presenter would stop talking, he would raise his hand and ask a question, extremely pertinent to what the presenter had said. As there was no preview of the data and nor any abstracts available to the participants, it was amazing that Claude was able to keep part of his brain awake, register the data and ask the key questions. In the ensuing years, this pattern was repeated all the time; and while by now I should be used to it, I am not; I am still surprised and amazed on how he does it.

Claude and I met in several international meetings, and we had the chance to socialize. In Erice (Sicily), we shared several meals with his wife Anne and my son Jared. We were invited to many meetings together such as Kindling 3, Kindling 4, Kindling 5, Kindling 6, and the biennial WONOEP meetings. During these meetings, there were some free evenings where we would all relax, have a drink, go dancing as we did in Salvador (Brazil) or sit around and tell jokes. In 1999, we were in Ceszky‐Krumlow a place where Egon Schiele did some of his influential work. Nevertheless, Ceszky‐Krumlow is a very sleepy town even now. No wonder that Egon Schiele had a hard time there. A group of us went down to the only bar/tavern after dinner in town. They served only mead and no food. We sat around telling jokes. Claude was the epicenter. His jokes on the behavior of Belgian people (he was the only one in the company to hold a license to comment on the Belgians) made us laugh so hard that the owner of the tavern asked us to leave because we bothered the regular customers. I think that was the only time that I was been kicked out of a tavern.

Claude has continued his extremely productive work on trying to understand the causes and the consequences of status epilepticus. While he has focused less on the developing brain, he has continued making pioneer observations and developing new treatments. He organized several conferences on status epilepticus, which were extremely well attended and generated healthy debates. Yet in the spirit of Claude, the discussions were polite and effective trying to find the common ground.

Claude is an icon in Epilepsy research. I am personally honored to have been his colleague and friend for over all these years. I learned from him how to be open minded, yet critical and to always reconsider data interpretation with a sense of humor.

*P.S*. Claude came to the premiere of Jared's first movie “Dead Man's Burden” (2012) in Los Angeles. Some bonds are strong.

## 4 A WORD FROM ASTRID NEHLIG, PHD

The first time I met Claude Wasterlain was in the mid‐80s. At that time, I was working in Nancy (France) as part of an INSERM (French Medical Research Institute) unit, whose basic/translational research was devoted to neonatal diseases, including hypoxia, seizures, and chronic exposure to antiseizure medications. We had decided to organize an international meeting in Nancy‐Pont‐à‐Mousson on “Neonatal Seizures: Pathophysiology and Pharmacological Management” and contacted Claude to be part of the scientific committee and main organizer of this meeting. I was quite junior at that time and not too familiar with clinical aspects of epilepsy. I was very impressed by Claude during the preparation of the different sessions of this meeting. Firstly, he would suggest many topics and even more impressive to me, he had a name for every single presentation of a given topic including many scientists and clinicians that I had not heard of at that time. In addition, Claude managed and helped to get a sponsorship from NIH, and we were very happy to have the double label INSERM/NIH for our meeting, which we would not have been able to manage without him. The meeting took place in September 1987 and again with Claude's help.

During our common work on that book and repeated meetings, Claude encouraged me to join WONOEP (Workshop on Neurobiology of Epilepsy). At that time, my work was not only centered on epileptic seizures, and I was not going to ILAE meetings and had never heard about WONOEP. Following Claude's advice, I applied for and attended the 2nd WONOEP meeting in Salvador (Brazil). And from then on, I attended most WONOEP meetings; I integrated into the epilepsy community and strengthened my collaboration and exchanges with Claude. I am very grateful to Claude to have encouraged me to become more active in the field of epilepsy where I met wonderful colleagues and was able to do very interesting work, at least in my eyes.

**FIGURE 3:**
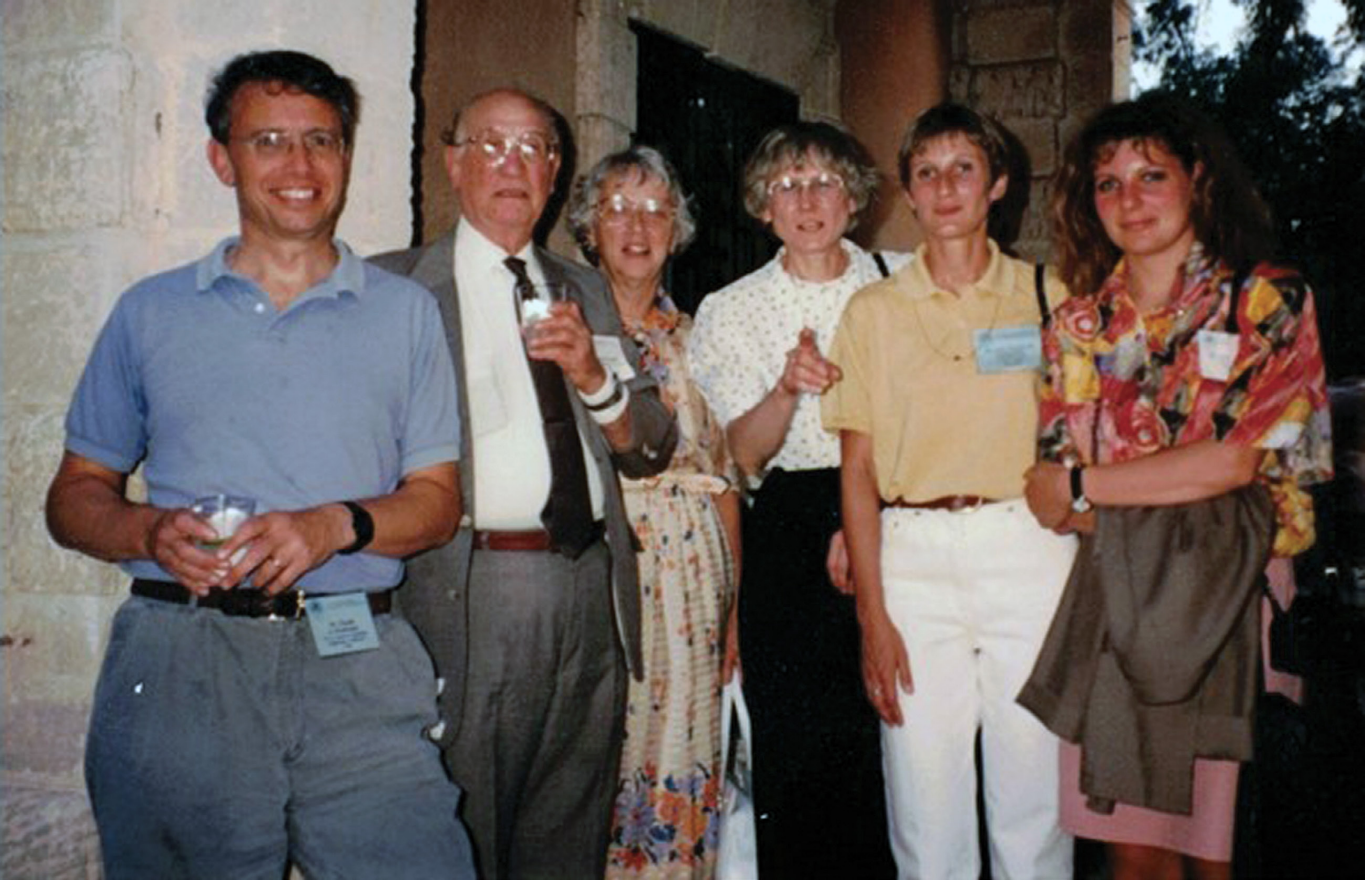
*Left to right*: Dr. Claude G. Wasterlain, Dr. Louis Sokoloff, Mrs. Louis Sokoloff, Dr. Astrid Nehlig and two of her students (names have been lost). XV International Meeting on Cerebral Blood Flow and Metabolism, Miami, Florida, 1991. *From the archive of Astrid Nehlig*.

**FIGURE 4:**
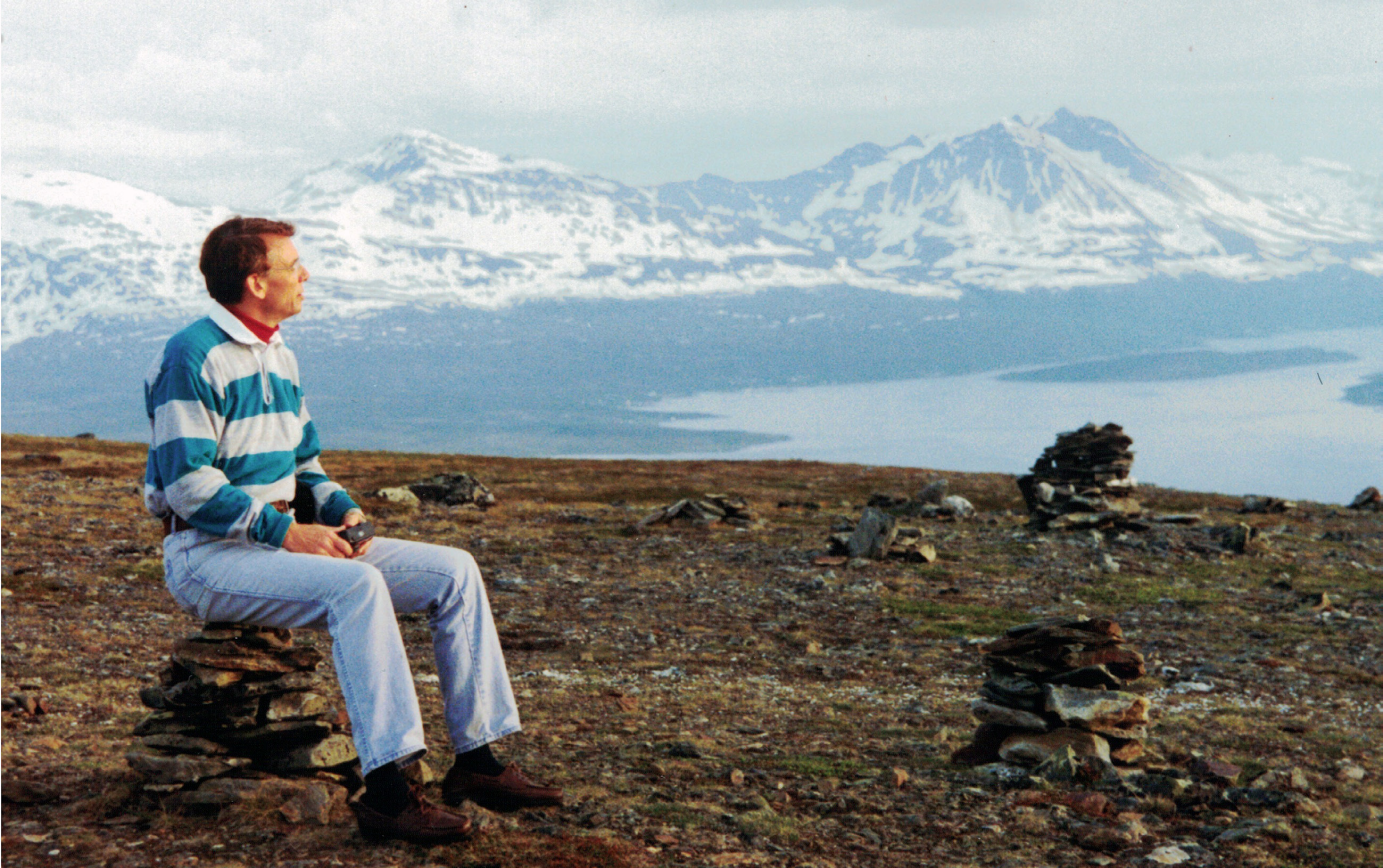
Dr. Claude G. Wasterlain, Tromsø, Norway, 1993. *From the archive of Solomon L. Moshé*.

I also knew Claude in another context. We used to meet every other year at the meetings of the International Society for Cerebral Blood Flow and Metabolism (Figure 3). At a time, when it was not a topic interesting too much the epileptic scientific community, both Claude and I were interested in cerebral metabolism and blood flow and their interrelationship/coupling during prolonged seizures. We raised the hypothesis that cerebral blood flow would not be able to sustain long enough the increased metabolic demand and that this uncoupling would be at least partly, leading to neuronal damage. We had a very fruitful collaboration on these issues, both in the adult and immature brain (Figure 4).

Finally, Claude became also one of the central participants in another meeting we organized in Obernai close to Strasbourg, France on “Childhood Epilepsies and Brain Development.” We were again able to gather most of the specialists of the topic and produced a very fruitful meeting.

Altogether, I would like to express all my admiration for Claude Wasterlain, both as a person and for his achievements. Apart from my more specific personal experience with him and from what I have briefly mentioned above, I would like to acknowledge his impressive level of scientific knowledge, both clinical and basic/translational. He has a high capacity of analyzing and integrating the available data and has constantly innovative ideas on multiple topics. In addition, he is a very charismatic person, helping young scientist/clinicians to find their way in epilepsy research.

In my eyes, Claude Wasterlain belongs to the handful of really remarkable persons with an incomparable stature whose contribution to epilepsy under various forms will be remembered for long. Claude, I would just say thank you for the person you are, for all you have achieved during your fruitful and productive carrier and I feel much honored to have been able to work with you and to consider you as a real friend.

## 5 CLAUDE G. WASTERLAIN, MD

### 5.1 Science, serendipity, and the sacred disease

Serendipity rules our lives. I wanted to be a writer, but my family convinced me that it was impossible to make a living by writing fiction, and that I should become a pharmacist and write on the side. Incidental exposure to pre‐medical courses showed them to be considerably less boring than the rest of my curriculum, so I switched majors. My curiosity was rewarded in much unexpected ways, and I found out what I wanted out of life.

The second patient I worked up in medical school had alexia without agraphia. She could write almost perfectly, but if her attention was diverted for a few seconds, she was unable to read the sentence that she had just written. She could name the letters individually but could not understand the meaning of written words. She also had great difficulty understanding the purposes of objects. I remember her describing a ballpoint pen: “It is round, long and made of metal. I do not know what it is”. She had no idea how to use it. To me, this illness seemed to open a window on how the brain works. It was so fascinating that I instantly gave up my plans to specialize in surgery and to practice in my hometown. I would become a neurologist and do brain research. To this day, I feel incredibly lucky for this chance encounter, and I have never regretted my decision.

#### 5.1.1 A reality check

A neuroradiologist named Andre Thibaut noticed my extensive work‐up in that patient's chart and told me that I should do research. Nothing was known about the reabsorption of cerebrospinal fluid (CSF), and if I came up with a project, he would get me a grant. I reviewed the literature, came up with a terrible idea, and he got me a grant. A Russian publication seemed to show that a large portion of CSF reabsorption was done through lymphatic vessels in the nose. Like most beginners, I did not understand how to design an experiment. This publication looked good to me, and I wanted to repeat it. I learned how to cannulate lymphatics, looked for a dye marker which had been injected into CSF. I did not find a lot of dye in nasal lymphatics but was too naïve to doubt the veracity of published data, and my intramural report concluded that I had confirmed my esteemed colleague's results, but instead of 32% of CSF reabsorbed through nasal lymphatics, under my conditions the percentage was 0.2%. Just to be sure, I also switched my marker to inulin, which avoided some of the pitfalls associated with dyes.^1^ Only later did I learn that under Trofim Lysenko, scientists in the Soviet Union had to either confirm their boss’ hypothesis or risk an extended vacation in Gulag. That experience taught me that my deference for anything that was published in a scientific journal had to be tempered by a healthy dose of skepticism. Show me the facts!

I learned from that mistake, and in my next experiment, I injected a radioactive neutral amino acid into the *cisterna magna*, froze the anesthetized rats in liquid nitrogen, cut the brain, and exposed the autoradiograms in liquid nitrogen for weeks, which produced accurate pictures of their reabsorption through the granulations of Paccioni on dural sinuses.^2^ That experiment later got me accepted into Fred Plum's Neurology program at Cornell, where I learned from Jerry Posner and Fred Plum how to design an experiment.

#### 5.1.2 Introduction to science

Before starting my residency, I began a research fellowship at Cornell, and Jerry Posner assigned me to a project on cerebral edema. The electron microscope had recently been invented, and two highly respected labs had used it to study cerebral edema, with opposite results. In one publication, swelling involved all cell types. In the other one, only astrocytes swelled, while neurons were anatomically normal. I was to induce edema with water intoxication, use newer fixatives and techniques and resolve the problem. When I told Jerry Posner that the paper that reported astrocytic swelling looked very solid, and that I wanted to model my experiments after it, he asked me whether reproducing it would unequivocally solve the problem. Needless to say, I repeated the other experiment. My lab could not afford to buy diamond knives, but I learned from Dick Torack, our neuropathologist, how to break glass in a very precise way to make glass knives sharp enough to cut brain tissue for electron microscopy and found that when I induced cerebral edema with water overload, only astrocytes swelled, protecting the neurons.^3^, ^4^

That experience solidified my commitment to research, and the atmosphere created by Fred Plum, Jerry Posner, Don Reis, and Paul McHugh showed me that clinical Neurology was exciting and that brain disease opened vast windows into brain function. If only we were smart enough, we could begin to understand how the brain works. I knew that I needed some serious basic science training if I wanted to be successful in that endeavor. Luckily, Cornell offered me a faculty position. It would be available after I completed my military service, which was then mandatory and universal. I could not get around a NATO agreement, which forced me to do it in my native Belgium, but I turned out to be the only person in the Belgian army who knew how to do cerebral angiograms, which had been a part of Neurology training in New York. That got me assigned to the military hospital in Brussels and gave me some spare time to go back to school. I studied molecular biology at the Free University of Brussels' Institute of Molecular Biology, where Brachet discovered the first clues to RNA's function.

#### 5.1.3 Science and serendipity lead to epilepsy

My passion at that time, shared with most neuroscientists of my generation, was molecular mechanism of memory. I wanted to work on Aplysia but could not find that mollusk in any research lab in the vicinity of Brussels. But the genetic code had just been deciphered, and the first eukaryotic messenger RNA (mRNA, the message for globin) had just been isolated in the lab that I joined. I would study brain mRNAs and their role in memory. As soon as I started isolating brain polyribosomes, the complex of mRNAs and the ribosomes, which translate it into proteins, it became evident that most of them coded for 40 000–50 000 molecular weight proteins and that the prevalent theory of that time was unlikely to be correct. If brain memories were modeled after immunological memories and consisted of modifications of large proteins, the messages for those large proteins were nowhere to be seen.^5^, ^6^

I needed a way to relate my findings to memory, and the most reproducible way to erase memories was seizure‐induced amnesia. When I started inducing electroconvulsive seizures in my rats, this resulted in a dramatic dissociation of polyribosomes, leaving mRNAs naked in the cytoplasm.^7^ I could not find much sign of degradation of that naked mRNA, so that was not the mechanism of amnesia. But seizures profoundly inhibited brain protein synthesis, even if oxygenation and blood pressure were maintained.^8^ We showed that this used an amplification mechanism in which a small seizure‐induced decrease in ATP generates a large increase in GDP which blocks the first step of translation^9^, ^10^ and may indeed play a role in amnesia.

When I came back to Cornell, Fred Plum was beginning a sabbatical year and Jerry Posner had moved to Memorial‐Sloan Kettering Cancer Center. They left idle a number of excellent technicians well trained in controlling metabolic components of seizures, and I started looking for the component of experimental seizures that caused amnesia. These experiments taught me a lot about epileptic seizures and their metabolic consequences. The results were highly relevant to problems I was encountering every day on the Neurology wards and changed the focus of my research to epilepsy.

Repeated convulsive seizures rapidly led to lactic acidosis and to death, which was delayed by correcting the acidosis.^11^ The first few seizures massively increased arterial blood pressure and cerebral blood flow (CBF), but if seizures continued in my animals, blood pressure and CBF fell, while cerebral metabolic rate remained quite high, resulting in a mismatch between energy supply and energy demand^12^, ^13^, ^14^, ^15^ and in selective neuronal loss.

Surprisingly, if seizures were repeated for over half an hour, in paralyzed, ventilated non‐convulsing rats without hypoxemia, acidosis, or hypotension, when stimulation stopped, seizures continued on their own.^11^ We had serendipitously developed the first model of self‐sustaining status epilepticus (SE), and it explained in part why established SE is so hard to stop. Later, we generated a less labor‐intensive model of self‐sustaining SE by intermittent stimulation of the perforant path in awake, free‐moving rats,^16^ and this enabled our lab to study the mechanisms involved.

#### 5.1.4 Status epilepticus

Trousseau stated that during SE, “something happens in the brain…that requires an explanation”.^17^ Like many observations by nineteenth‐century pioneers, this proved correct. In our rat model of SE, repeated seizures induced internalization and inactivation of many synaptic GABA‐A receptors,^18^ leading to failure of inhibition and to an increase in synaptic NMDA receptors,^19^ ultimately leading to a runaway excitation. AMPA receptors also moved to synapses^20^ and this move was NMDA receptor‐dependent. Extrasynaptic GABA‐A receptors, however, did not internalize and remained active after an hour of continuous SE.^18^ This hippocampal circuit is strongly modulated by neuropeptides, including galanin,^21^ tachykinins,^22^ and opioids.^23^

Therapeutic implications of these changes in receptor trafficking seemed obvious. First, in the presence of both failure of inhibition and runaway excitation, why treat only one of them? Current guidelines for the initial treatment of SE recommend monotherapy with a benzodiazepine. This GABA‐A agonist is effective when given early in the course of SE, but rapidly loses potency as synaptic GABA‐A receptors internalize, and this treatment leaves the changes in glutamate receptors untreated. Indeed, in our animal models, a combination of a benzodiazepine with the NMDA antagonists ketamine or dizocilpine is synergistic^24^ and easily terminates benzodiazepine‐refractory SE.^25^

Second, when treatment of SE is delayed, benzodiazepine potency falls, and there may not be enough GABA‐A receptors left in synapses to restore inhibition. In order to stop SE, it may be necessary to add a third drug to the benzodiazepine/ketamine combination, in order to reduce excitation and/or enhance inhibition at a non‐benzodiazepine site.^26^ Furthermore, since receptor trafficking is time‐ and seizure burden‐dependent, giving drug combinations early and simultaneously is far more effective than giving the same drugs at the same dose sequentially.

Third, we may be able to take advantage of the lack of internalization of extrasynaptic GABA‐A receptors by using drugs that target those receptors, such as neurosteroids.^18^

#### 5.1.5 Seizure‐induced epileptogenesis

When Graham Goddard developed the kindling model of epilepsy, it connected my interests in memory and in epilepsy. We induced kindling with muscarinic agonists and showed that the activation of muscarinic synapses was necessary and sufficient to induce kindling, since mirror‐image stereoisomers failed to elicit kindling.^27^ In other words, repeated activation of a group of muscarinic synapses was sufficient to induce chronic epilepsy. We also showed that calmodulin kinase II was intimately involved in kindling,^28^ and a later discovery of its role in long‐term potentiation confirmed the link between kindling and memory mechanisms. As usual, disease uses molecules and circuits involved in normal brain function to do its deed. In this case, kindling stimulates the wrong group of synapses at the wrong time and uses the pre‐existing biochemistry that normally subserves memory to generate chronic epilepsy.

#### 5.1.6 Seizures and brain development

Here again, our work led us back from the study of amnesia to the study of epilepsy. We found that seizures severely inhibit brain protein synthesis in vivo, even in the absence of hypoxemia, hypotension, and other major metabolic disturbances.^8^, ^29^ At that time, our Cornell lab neighbor Myron Winick was studying effects of malnutrition on the brain.^30^ He showed that brain protein synthesis was selectively protected from the effects of malnutrition, but if malnutrition was sufficiently severe and prolonged during critical periods, development was irreversibly impaired. Bypassed developmental steps could not be retraced. This seemed highly relevant to the prolonged inhibition of protein synthesis that my lab found after seizures in immature animals. We were surprised to find that very little experimental work had been done on the consequences of seizures on brain development, although a significant clinical literature on that topic had been published.

We started giving repeated seizure to rat pups, selecting seizure models that caused no histologically visible neuronal injury. The results varied dramatically with the stage of brain development at the time of seizures. Both single seizures administered three times a day for 10 days or a single bout of status epilepticus at postnatal Day 4 delayed developmental milestones and impaired brain growth.^31^, ^32^ This was not due to anoxemia or malnutrition.^33^ Seizures during the immediate postnatal period, a time of very active cell division in the rat brain, curtailed cell numbers. Seizures between Days 9 and 18, when cell differentiation is the dominant process, did not alter cell numbers but may have curtailed cell‐to‐cell connections, since they reduced synaptic markers and myelin accumulation.^34^, ^35^ Seizures after postnatal Day 19 altered neither. Later studies showed that the vulnerability of specific neuronal populations depended in part on their maturational level at the time of the seizures.^36^

Finding behavioral and anatomical sequelae of relatively mild seizures in rodents raised important questions about the effect of seizures on human brain development. At that time, seizures in neonates and infants were thought to be relatively benign and were often not treated vigorously. When I presented our results at a pediatric meeting, I was shocked to find out how angry pediatricians could get, although overall they are probably the nicest and most serene group of people on the surface of the Earth. The controversy on the clinical relevance of these results lasted a number of years.^37^, ^38^ Other investigators produced elegant examples of the long‐term sequelae of seizures in the immature brain, such as the alteration of spatial maps of hippocampal place cells by seizures occurring during their maturation.^39^ Subtle developmental changes induced by seizures are now recognized as an important aspect of epilepsy in the immature brain.^40^

By design, our initial experiments produced no histological sign of neuronal injury. A number of prominent laboratories found little or no neuronal death in rat pups after seizures of a duration (but not severity) which would easily cause neuronal death in mature animals.^41^, ^42^, ^43^ This gave rise to a separate controversy: are immature neurons impervious to seizure‐induced injury? Because of low metabolic rate in the immature brain, neuronal injury takes longer to develop; but can it still occur if seizures are sufficiently severe and prolonged? This is a critical question for prolonged febrile seizures and their role in the etiology of hippocampal sclerosis and temporal lobe epilepsy. Our studies showed that seizures at postnatal Day 10 severely damage the rabbit hippocampus,^44^ that seizures induced by perforant path stimulation at postnatal Day 14–15 in rats cause widespread neuronal injury^45^ and that seizures induced by lithium‐pilocarpine caused widespread neuronal injury in brain regions which vary with age at the time of seizures.^46^ Later studies showed that seizure‐induced neuronal injury occurs as early as in postnatal Day 7 rat pups.^47^ Within the same brain region, the maturation stage of individual neuronal populations is a key component of their vulnerability and of the apoptotic versus necrotic type of cell death.^48^ MRI studies of febrile status epilepticus have shown hippocampal swelling followed by atrophy evolving into hippocampal sclerosis,^49^ supporting the view that some types of prolonged seizures in humans may also lead to neuronal injury and its long‐term consequences.

#### 5.1.7 The end

My journey from clinical observation to basic research into mechanisms of brain function and brain disease has been exciting and fulfilling. I was lucky that serendipity directed me to study what McDonald Critchley called “the divine banquet of the brain.” I was most fortunate to have shared that journey with a number of talented collaborators and to have had the opportunity to contribute to the scientific development of many students and young investigators who kept me curious and excited until my twilight years. I feel privileged to have been a minor participant in the greatest age of medical discovery the world has ever known. My clinical and scientific mentors Jerry Posner and Fred Plum set the tone by introducing me to clinical neurology as a branch of science, and to science as the key to the future of mankind. To be meaningful, individual contributions need not be either highly publicized or widely recognized. The pursuit of objective knowledge using tools of reason will make our world more compassionate and more humane, will eventually cure disease and bring us together, even if there are many temporary setbacks on the way.

## CONFLICT OF INTEREST STATEMENT

The authors have no conflicts to disclose. CGW was supported by research grants from the Veterans Health Administration, NIH/NINCDS, the American Epilepsy Society, the Epilepsy Foundation of America, the American Heart Association, the James and Debbie Cho Foundation, Wallace Laboratories, Inc., UCB, Inc., Johnson and Johnson, Inc., and Eisai, Inc. These activities do not constitute conflict of interest with the reference to this article.

## ETHICS STATEMENT

We confirm that we have read the Journal's position on issues involved in ethical publication and affirm that this report is consistent with those guidelines.
